# Focusing by shape change in the lens of the eye: a commentary on Young (1801) ‘On the mechanism of the eye’

**DOI:** 10.1098/rstb.2014.0308

**Published:** 2015-04-19

**Authors:** Michael Land

**Affiliations:** School of Life Sciences, University of Sussex, Brighton BN1 9QG, UK

**Keywords:** eye, optometer, lens curvature, cornea, accommodation

## Abstract

In his Bakerian Lecture paper of 1801, Thomas Young provided the best account up to that time of the eye's optical system, including refraction by the cornea and the surfaces of the lens. He built a device, an optometer, for determining the eye's state of focus, making it possible to prescribe appropriate correction lenses. His main contribution, however, was to show that accommodation, the eye's focusing mechanism, was not the result of changes to the curvature of the cornea, nor to the length of the eye, but was due entirely to changes in the shape of the lens, which he described with impressive accuracy. He was wrong, however, in believing that the reason the lens bulges when focusing on near objects was because it behaved as a contracting muscle. Half a century later, Helmholtz showed that the lens bulges not by its own contraction, but when it is relaxed as a result of contraction of newly discovered circular muscles in the ciliary body. This commentary was written to celebrate the 350th anniversary of the journal *Philosophical Transactions of the Royal Society*.

## 1. Introduction

A recent biography of Thomas Young has an arresting title*:* ‘The last man who knew everything: Thomas Young, the anonymous polymath who proved Newton wrong, explained how we see, cured the sick and deciphered the Rosetta Stone′ [1]. Hermann von Helmholtz is the only other nineteenth century scientific polymath who can be compared with Young, and intriguingly they both tackled similar subjects. Helmholtz built on Young's trichromacy theory, in which Young had shown that the classic Newtonian rainbow colours could all be produced by suitable combinations of lights of not more than three hues [2]; Helmholtz′ contribution was to define, in 1850, the spectral composition of these basic hues [3], and his findings, together with those of Young, form the universally accepted Young–Helmholtz trichromacy theory of colour vision. The three colour mechanisms it embodies were matched by measurements of the spectral absorption of the three kinds of retinal cones, but not until 1980, well over a century later [4]. The other area where Helmholtz took up a theme pioneered by Young was the mechanism of accommodation of the eye. Here the story is more interesting. Young had demonstrated that changing focus must be the result of curvature changes in the surfaces of the lens, but then, for entirely understandable reasons, proposed the wrong mechanism. Helmholtz provided an alternative account, which remains the standard explanation today [5]. I will explore this in more detail later. Young's other great achievements—the experimental demonstration that light is a wave motion, notwithstanding Newton's hostility from beyond the grave, the researches into elasticity which are remembered in the measure known as ‘Young's Modulus′, and the partial decoding of the script of the Rosetta Stone—are all formidable achievements, but are beyond the scope of this article. On top of all this, Young was a physician with a practice in London, and it was to maintain his reputation as a physician that he published some of his first scientific articles anonymously. He maintained his medical work throughout his scientific career. Interestingly, both Young and Helmholtz first trained as medics, and learnt their physics afterwards.

## 2. The Bakerian Lecture 1800

Young gave the Bakerian Lecture, a prize lecture of the Royal Society, in 1800, 1801 and 1803. The first of these, entitled ‘On the Mechanism of the Eye′ was presented in November 1800 and published in the journal in 1801 [6]. It is a survey of Young's many studies of the workings of the eye. The lecture is in four main parts. A brief introduction is followed (p. 27) by a section on ‘Dioptrical Propositions′ in which he develops some of the optical theory that he uses later to model the optics of the eye. He then (p. 33) describes an instrument, an ‘optometer′, for determining the location of the eye's best external focus, and so ‘is easily applicable to the purpose of ascertaining the focal length of spectacles required for myopic or presbyopic eyes′. He then develops an optical model of the eye (p. 38) based on the best measurements of surface curvatures and refractive indices available: the image surfaces produced by this model are shown in his fig. 16 (The original figures are provided as supplementary material.). Most of the rest of the lecture (p. 51 onwards) is devoted to the problem of accommodation: how the eye adjusts its focus to objects at different distances. This is the main substance of the paper, for which the earlier sections are almost preliminaries. A recent, more extensive, account of the lecture is given by Atchison & Charman [7]. Students or other readers who would like a brief introduction to the anatomy and physiology of the eye may find my own short book useful [8].

## 3. Dioptrical propositions

By modern standard this section of the paper is dauntingly formal, with propositions, scholia and corollaries. This was the standard format for mathematical treatises at the time. The first six propositions are more or less straightforward extensions of Newtonian optics to specific problems of refraction at surfaces in the eye, but propositions VII and VIII are different and, in Young's words ‘require a long demonstration′, which is not part of the lecture. The problem, in proposition VII, is ‘To find the principal focus of a sphere, or lens, of which the internal parts are more dense than the external′: in other words, an optically inhomogeneous or graded-index lens. Young was aware that the human lens was denser in the centre, and this proposition provided a way to find the focal length of such a lens, if the gradient of refraction within it was known. Unfortunately, the description here is too brief to follow in detail, and not all the relevant variables are defined. Helmholtz had trouble with Young's workings too [4, p. 107]: ‘… on account of its conciseness it is often hard to follow, and, moreover, it presupposes the most thorough knowledge of mathematical optics′. I am relieved to find myself in such good company. Nonetheless, Young's was the first attempt to work out the refracting power of an inhomogeneous lens, and it was not until 1944 that an explicit solution to the form of the gradient in such a lens, free from spherical aberration, was finally determined [9]. Young's final proposition (VIII) is an extension of the logic of VII to oblique rays, and is even more abbreviated and obscure.

Part of the problem of trying to understand Young's optics is that he does not have a single value for the refractive index of a substance, but uses—as Newton did—the ratios of sines of angles at each interface, in accordance with Snell's law. Thus, in proposition I he states that the refraction of air into water is 4 to 3 (i.e. 1.33, which is indeed the refractive index of water as we would understand it), but that the refraction of air into glass is nearly that of 3 to 2 (1.5, also a good approximation). So air is identified by the number 3 in one case and 2 in the other, which makes it difficult for a modern scientist to follow Young's subsequent logic. In fact Young himself invented the term ‘index of refraction′ and used it as a single figure in the modern sense, but not until 1807 [10, p. 413], too late for the 1801 lecture.

## 4. The optometer

Partly to further his investigations into accommodation, Young devised an optometer: an instrument for determining the focal distance of the eye. Eyes of younger people have a range of distances, between a far point and a near point, over which objects can be brought to a sharp focus. In normal sighted (emmetropic) individuals, the far point is at infinity, but the near point recedes with age until reading normal print becomes impossible. Usually, this condition (presbyopia) sets in by the age of about 45, by which time the lens has lost its elasticity, and glasses then become essential for close work. At a time when doctors were unlikely to keep a stock of trial lenses, an instrument for measuring an individual's range of focus could provide a way of determining the strength of glasses required. This also applies to short sight (myopia) and long sight (hyperopia) where the far point is beyond infinity.

**Figure 1.**
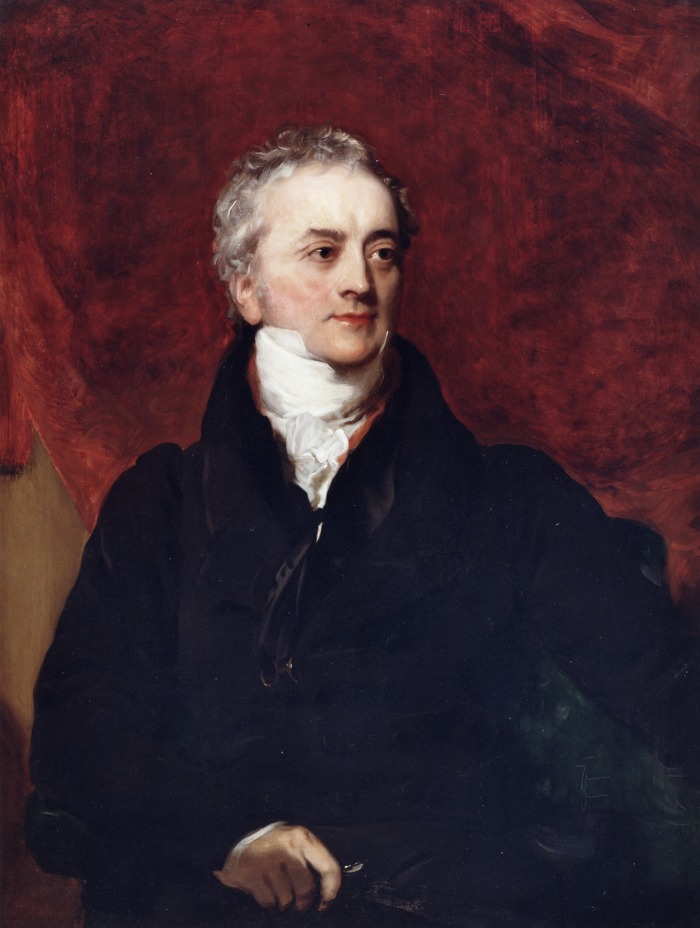
Portrait of Thomas Young by Henry Briggs *ca* 1822. Young was fairly myopic, and is holding his glasses in his hand, no doubt deliberately. Copyright © The Royal Society.

**Figure 2.**
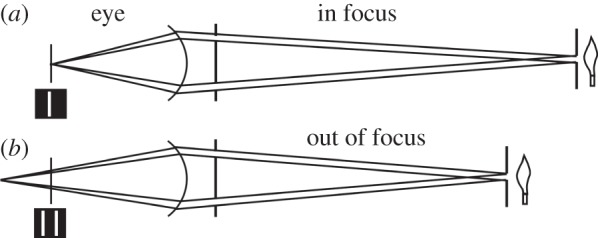
The principle of the optometer. An image of an illuminated slit, seen through two slits close to the eye, will have a single image on the retina (*a*) when the slit is in focus. When out of focus (*b*) there will be two images on the retina (insets left). The distances are not to scale. Based on Young [6, fig. 5]. Young's instrument used a horizontal line rather than a vertical slit (see text).

Young's optometer was a modification of an earlier instrument invented by William Porterfield in 1738 [11], and the principle goes back to an observation by Christoph Scheiner in 1619 [12]. Scheiner had noticed that when a card with two or more pinholes in it was held in front of the eye, multiple images were seen if the illuminating candle was not in focus on the retina (figure 2). Porterfield's instrument exploited this phenomenon using slits rather than pinholes. A card with two narrow closely spaced vertical slits is held in front of the eye so that both are within the limits of the pupil. These are illuminated by light from a single third slit in front of a light box, situated at a distance from the eye corresponding to where the eye might be expected to focus (figure 2). Then, if the eye is indeed focused on the illuminated slit, its image will be seen on the retina as a single vertical line. If, however, the eye is focused beyond the slit, then the image of the slit will fall behind the retina. The two slits that allow light into the eye produce separate narrow ray-paths within the eye, so that when these paths meet the retina they will be seen as separated lines. The distance of the illuminated slit from the eye is then adjusted until a single image is restored; this is then the eye's focal distance. In his instrument, instead of the single target slit, Young used a line stretching away from the eye slightly below sight level (illustrated in [7, fig. 1]). The point on the line that is in focus is imaged as a point on the retina, but the parts of the line in front of and behind this point form V shapes which meet in a cross. A slider on the line is then used to identify the crossing point. This can be repeated with the eye accommodating, or with lenses in the pathway, to establish the range of useful vision. Young's particular contribution was to equip the instrument with a scale, from which the distance of the focus on the line could be read off directly as the focal length of the lens required to correct for the eye's deficiency.

Young of course used the instrument on himself, with interesting results. He was myopic and astigmatic. ‘My eye, in a state of relaxation, collects to a focus on the retina, those rays which diverge vertically from an object at a distance of ten inches from the cornea, and in the rays which diverge horizontally from an object at seven inches distance’. (p. 39). This makes him, in modern terms, about four dioptres myopic with two dioptres of astigmatism. Young would have worn concave glasses for distant vision (figure 1), but at that time part-cylindrical lenses to cope with astigmatism could not be produced. His solution was to mount the spectacle lenses obliquely by tilting them by about 10°. The misalignment of the lens axis induces astigmatism of its own, and this could then be used to counter the astigmatism of the eye itself. Helmholtz believed this was the first written account of astigmatism [5, p. 199], although Young mentions that other practitioners had also found the use of tilted spectacles to be helpful in similar circumstances. Young's optometer was ‘subjective′, relying on the subject's report of what they saw. Modern optometers usually use an objective method in which an automated mechanism measures the position of best focus by imaging the retina directly.

## 5. The model eye

In fig. 16 of the lecture, Young gives us an optical diagram of the eye, showing the form and location of the image shells produced by each optical surface in turn. The first image from the cornea is well behind the retina, then as the contributions from the anterior and posterior surfaces of the lens are added, the image shells contract towards the retinal surface. The final surface, incorporating all refractions, he shows to coincide closely with the retina itself (see also [5, fig. 6]). Young used the best estimates of refractive indices and curvatures available at the time, including some measurements made on his own eye. He does not incorporate the inhomogeneity of the lens into his final calculation, but ‘if the law, by which the density varies, were more accurately ascertained … probably the image, thus corrected, would approach very nearly to the form of the twelfth curve′ (i.e. the retina).

Young's model eye was by no means the first. The basic optical structure of the eye had been known since Johannes Kepler in 1604, and in 1652, a century and a half before Young, Christiaan Huygens [13] had described a ‘simplified eye′ consisting of a single spherical refracting surface separating air from water, throwing an image onto another spherical surface with three time the radius. Given that the back of the eye has a radius of about 11 mm, this will give a focal length close to 15 mm, which is a fair approximation to the currently accepted focal length (16.2 mm), and would have been quite adequate for working out the size on the retina of the image of an external object. Annoyingly, Young does not provide a scale for his model eye in his fig. 16, and is reticent about providing a definite figure for the focal length of the eye, but he has his reasons: ‘it is very difficult to ascertain the proportions of the eye so exactly as to determine, with certainty, the size of an image on the retina′ (p. 48). However, on p. 49, as a result of an experiment rather than a calculation, he does come up with a distance from the intersection of the principal rays (i.e. the nodal point) to the retina of ‘637 thousandths′. Thanks in some measure to our then enemy Napoleon, this translates from inches to 16.18 mm, a figure that couldn't be any better. More recent studies, by Listing [14] and particularly by Gullstrand [15], have barely improved upon Young's model [7, pp. 3–6, fig. 2].

**Figure 3.**
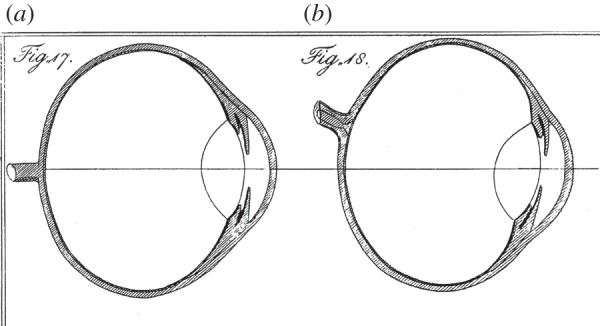
Young's illustrations of vertical (*a*) and horizontal (*b*) sections through his eye [6]. ‘I have endeavoured to express in four figures, the form of every part of my eye, as nearly as I have been able to ascertain it′. His figs. 19 and 20 show front views, in different states of pupil dilation. Copyright © The Royal Society.

In this section, Young provides diagrams of his own eye (figure 3), and he also deals with the eye's field of view, the location of the blind spot and chromatic aberration. But we must move on.

## 6. Accommodation

Young is justly famous for very nearly sorting out how the eye focuses on objects at different distances. The mechanism he came up with was wrong—he proposed that the lens behaved as a muscle—but the studies he made leading up to this conclusion were, in all but the last step, faultless. There are many possible ways that accommodation might come about: the curvature of the cornea might change, altering its focal length; the eyeball might change in length, so altering the distance of the retina from the lens; or the lens itself might change its shape, and hence its focal length. Young worked systematically through each possibility, convincingly eliminating the first two.

He calculated that, for the cornea to produce the change of focus he measured in his own myopic eye (from 10 inches to 29 tenths), its radius ‘must be diminished from 31 to 25 hundredths′. Such a change of radius would considerably alter the size of images reflected from the cornea. Young devised various ways of viewing the reflected images of candles and scales of different kinds in his own eye and those of others, while at rest and when accommodating. He was the ideal subject for this: ‘I must remark that, by a little habit, I have acquired a ready command over the accommodation of my eye′. Whatever he did, he was unable to detect any changes in the sizes or separation of the reflected images in the cornea. Had the required changes in radius that he had calculated actually occurred, he would have had no difficulty in detecting them. He satisfied himself that ‘the cornea is not concerned in the accommodation of the eye′.

Changes in the length of the eyeball were more of a challenge to measure. His calculations showed that the change in distance from lens to retina, required to cover his range of accommodation, was ‘an elongation of 135 thousandths, or more than one-seventh of the diameter of the eye′. Measuring the length of the eye *in situ* is not practicable, but it might be possible to detect any consequent change in diameter. Young's method was to prevent a change in the diameter of the eye, and see whether this affected accommodation. His technique is not for the squeamish. ‘Another test … was the application of the ring of a key to the external angle, when the eye was turned as much inwards as possible, and confined at the same time by a strong oval iron ring, pressed against it at the internal angle. The key was forced in as far as the sensibility of the integuments would admit, and was wedged, by a moderate pressure, between the eye and bone′. I have no desire to repeat this procedure, but will take Young's word for it that ‘the elongation of the eye must have been either totally or very nearly prevented′. Atchison & Charman [7, p. 8] are somewhat sceptical on this point. However, the outcome was that accommodation was in no way impeded. In another experiment he arranged for the images of two candles to straddle the edges of the blind spot, expecting that if there were an elongation of the eye's axis when the eye accommodated ‘the external candle would appear to recede outwards upon the visible space. But this did not happen′. He concludes: ‘it appears to be highly improbable that any material change in the length of the axis actually takes place′. Realistically, this only leaves a change in the shape of the crystalline lens as the basis for accommodation, an idea originally proposed by Descartes in 1637.

Before considering what changes in lens curvatures would be needed, Young had first to address a longstanding objection to the idea itself, namely the claim that patients who had a lens removed still had some power of accommodation. Through the good offices of a practitioner friend, Mr. Ware, Young was able to examine a number of patients who, for various reasons, had had one or both lenses removed. All these patients wore glasses to compensate, and all had a modest depth range over which they could distinguish letters. However, when tested on the optometer (figure 2) , they all saw a single image, as opposed to multiple images, at one distance only, which indicates that their eyes had no power of accommodation. Their apparent ability to see at different distances was to be expected if they made the best use of adequate, though imperfect, images. In much the same way, someone with uncorrected presbyopia can still read out-of-focus large print.

**Figure 4.**
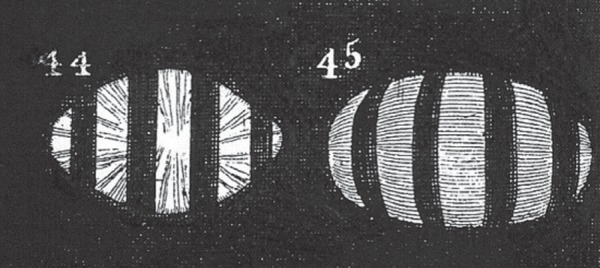
Young's figures of the appearance of four lines, viewed in the optometer (figure 2), with the eye relaxed [6, fig. 44] and accommodated [6, fig. 45]. ‘If the refraction of the lens remained the same, it is absolutely impossible that any change in the distance of the retina should produce a curvature in those shadows, which, in the relaxed state are found to be in all parts straight′ [6, p. 69]. Copyright © The Royal Society.

The most telling evidence in favour of a change in lens curvature during accommodation came from the appearance of the lines seen in the optometer when a grating was used in front of the pupil (figure 2). When the grating was illuminated by sources at various distances this produced a grid pattern on the retina. In the relaxed eye, these lines were straight, but when the eye accommodated they became curved, convex outwards, with the curvature greatest for lines furthest from the centre (figure 4). ‘The same appearances are equally observable, when the effect of the cornea is removed by immersion in water; and the only imaginable way of accounting for the diversity, is to suppose that the central parts of the lens acquire a greater degree of curvature than the marginal parts'. This is indeed incontrovertible evidence.

**Figure 5.**
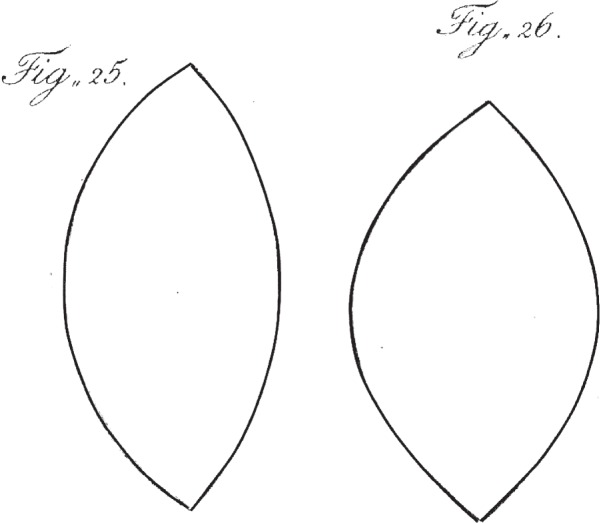
Young's illustration of changes in the form of the lens [6]. The anterior faces are to the right. ‘The form of the lens thus changed [by accommodation] will be nearly that of fig. 26; the relaxed state being nearly as represented in fig. 25’. Young was correct about the changes, but not in supposing that fig. 25 represents the relaxed state of the lens. Copyright © The Royal Society.

Young goes on to use the theory of lateral aberrations, outlined in his initial Dioptrical propositions, to interpret the curvatures of the grating, together with the results of some other experiments with the optometer, to work out the extent and form of the changes in the lens. The argument here is condensed and difficult, but the conclusions are astonishingly bold. ‘Here the anterior surface must be a portion of a hyperbola, … and the posterior surface must be nearly parabolical′. He illustrates the form of the lens in the eye's relaxed and accommodated states (figure 5).

**Figure 6.**
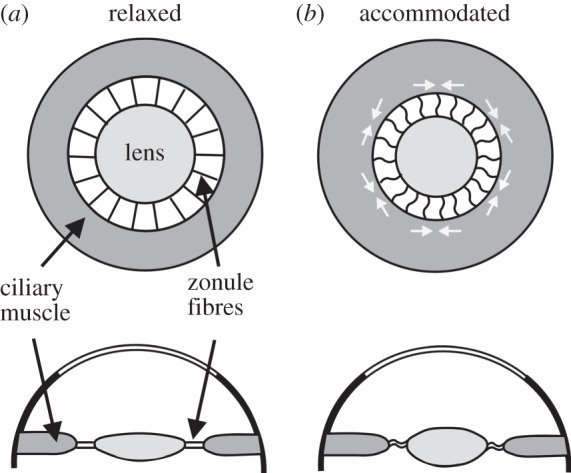
Post-Helmholtz view of accommodation. In the relaxed eye the lens is held, flattened, under tension by the zonule fibres (*a*). Contraction of the circular fibres in the ciliary muscle decreases the inner diameter of the ciliary body, releasing tension in the zonule fibres, and so allowing the lens to bulge as a result of the elasticity of its capsule (*b*).

This is where the problems begin. In terms of the modern (post-Helmholtz) interpretation of accommodation, Young's figures are the wrong way round. The flattened form of the *lens* in the relaxed *eye* is actually the one under tension, and the bulging lens of the accommodated eye is in its relaxed state. This would have seemed quite counterintuitive to Young; accommodation is clearly effortful, and so the lens in this state must be under tension. The lens is attached to the ciliary processes which surround it, but if these are contractile (figure 6), and pull radially, they will tend to flatten the lens, which is not what happens in accommodation. In 1793, Young & Brocklesby [16] had proposed that the lens changes to a rounder shape by the squeezing effect of external coats of contractile membranous tendons. In the present essay Young abandons this idea, but goes one stage further, and proposes that the lens *itself* is a muscle. His reason is simply that: ‘A muscle never contracts, without at the same time swelling laterally′. Again this was not a new idea, and Young attributes it to Dr Pemberton in 1719. The kind of histology that was available by the mid-nineteenth century would probably have disabused Young of thinking that the lens had the microstructure of a muscle, but in 1801 this would have seemed perfectly reasonable.

It was known at the time that muscles contract when stimulated electrically. Young made some experiments to see whether he could, by electrical means, induce contractions in a lens, presumably excised from a cow or sheep eye: ‘With this apparatus I made some experiments, assisted by Mr Wilkinson, whose residence was near a slaughter-house: but we could obtain, by this method, no satisfactory evidence of the change′. Others had also tried to evoke electrical contractions, but had similarly failed. A muscle must also have a nerve supply, and Young spent much time looking for it. ‘I have laboured with the most obstinate perseverance to trace nerves into the lens, and I have sometimes imagined that I had succeeded; but I cannot positively go further than to state my full conviction of their existence, and of the precipitancy of those who have absolutely denied it′. And later: ‘Our inability to discover them, is scarcely an argument against their existence′. One really feels for Young at this point. He seems to be trapped by a theory which must have seemed the only possible solution at the time. The next few pages are an interesting comparative survey of the eyes of other vertebrates, and even dragonflies, looking for other evidence of muscularity in their optical systems, but little comes out of it. Young had succeeded admirably in demonstrating that accommodation occurs entirely as a result of changes to the curvature of the lens, an extremely important contribution to our understanding of vision. It was, however, another half century before the mechanism of this change in lens shape came to be understood.

## 7. Helmholtz and accommodation

Hermann von Helmholtz (1821–1884) was one of the intellectual giants of the nineteenth century. His main contributions were in physics, but with a strong leaning towards physiology. His most famous books were *On the Sensations of Tone* (1862) and the *Handbook of Physiological Optics* (1855–1866), the third edition of the latter being translated into English by the Optical Society of America in 1924 [5]. Helmholtz wrote on many other subjects, often crossing the divide between science and art, as, for example, in his essay on ‘The relation of optics to painting′ in his *Popular Scientific Lectures* [17]. In his work on optics he took up the question of the accommodation of the eye, and solved the conundrum that had frustrated Young. By what muscular action can the lens increase its curvature, without actually being a muscle itself?

Helmholtz′ insight was to realize that it is the *accommodated* lens that is relaxed. ‘If the pull of the zonule [which attaches the lens to the surrounding ciliary body] is relaxed in accommodating for near vision, the equatorial diameter of the lens will diminish, and the lens will get thicker in the middle, both surfaces becoming more curved′ [5, p. 151]. That the lens capsule is elastic was known to Young, but he thought that the elasticity would return the lens to its unaccommodated—less curved—shape. But how is activation of the ciliary muscle able to result in relaxation of the lens capsule? The crucial information was provided in 1855 by van Reeken [18], and then by others. The ciliary body contains not only meridional (radial) muscle fibres but also circular ones. ‘The circular fibres of the muscle must pull the corresponding edge of the muscle towards the tip of the ciliary processes and towards the edge of the lens′ [5, p. 170]. In other words, the ciliary muscle can act as a sphincter, whose diameter reduces when stimulated, so removing the tension from the lens capsule, allowing the lens to bulge (figure 6). When viewing distant objects with the eye relaxed, the circular fibres of the ciliary muscle are not active, and the lens capsule again becomes stretched by the passive tension of the zonule fibres. This mechanism was endorsed by nearly all later students of the eye, and it is now the standard textbook account of accommodation. There have, however, been two challenges to the Helmholtz model in the twentieth century. Tscherning [19] suggested that the zonule fibres compressed the lens rather than relaxing it, and Schachar [20] has proposed that outward tension from the zonule fibres around the lens equator could flatten the outer zones of the lens, causing the centre to bulge, in the manner of a squeezed balloon. However, mechanical modelling, based on the most recent curvature data [21], has come down firmly in favour of the Helmholtz model, in which the curvature changes in the lens are the result of relaxation of the zonule.

Helmholtz recognized Young's pioneering work, and indeed had a very high opinion of him. It is interesting to read his assessment of Young, which gives some insight into the way Young's work was regarded in his own lifetime.

‘He was one of the most acute men who ever lived, but had the misfortune to be too far in advance of his contemporaries. They looked on him with astonishment, but could not follow his bold speculations, and thus a mass of his most important thoughts remained buried and forgotten in the ‘Transactions of the Royal Society’, until a later generation by slow degrees arrived at the rediscovery of his discoveries, and came to appreciate the force of his arguments and the accuracy of his conclusions′ [17, p. 133].

## Supplementary Material

Figures from Young, 1801

## Author profile

**Michael Land** is Emeritus Professor of Neurobiology at the University of Sussex. The first half of his research career was concerned with the eyes and vision of animals, which included animals from most phyla. The most important findings were that scallops and shrimps both use mirrors to form images, and that jumping spiders, some sea snails and mantis shrimps all have linear retinas with which they scan the surroundings. Since about 1990 he has mainly been interested in human eye movement strategies, during tasks such as driving, playing ball games, reading music and preparing food. The main conclusions have been that eye movements are driven by the needs of the task being undertaken, rather than the salience of objects, and that the eyes anticipate actions by up to a second. He was elected to the Royal Society in 1982, and received the Frink Medal of The Zoological Society of London in 1994. He has written three books and approximately 150 scientific papers.
