# Supplementary material for: Focusing by shape change in the lens of the eye: a commentary on Young (1801) ‘On the mechanism of the eye’
Source: Philos Trans R Soc Lond B Biol Sci. 2015 Apr 19;370(1666):20140308. doi: 10.1098/rstb.2014.0308 (PMC4360117; doi:10.1098/rstb.2014.0308)
Supplement: Figures from Young, 1801 [file rstb20140308supp1.pdf]

PHILOSOPHICAL
TRANSACTIONS:

The Bakerian Lecture: On the Mechanism of the Eye

Thomas Young

Phil. Trans. R. Soc. Lond. 1801 **91**, doi: 10.1098/rstl.1801.0004, published 1 January 1801

References

Article cited in:
http://rstl.royalsocietypublishing.org/content/91/23.citation#related-urls

Email alerting service

Receive free email alerts when new articles cite this article - sign up in the box at the top right-hand corner of the article or click **here**

To subscribe to *Phil. Trans. R. Soc. Lond.* go to:
http://rstl.royalsocietypublishing.org/subscriptions

Fig. 1.

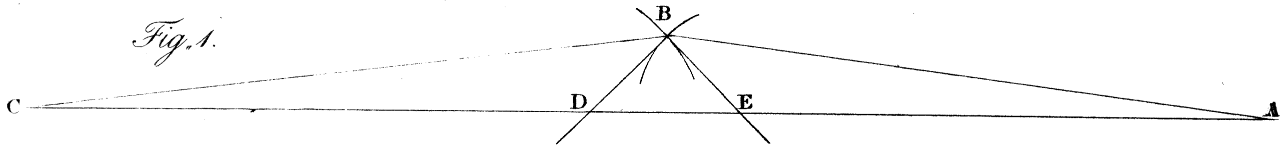

Fig. 2.

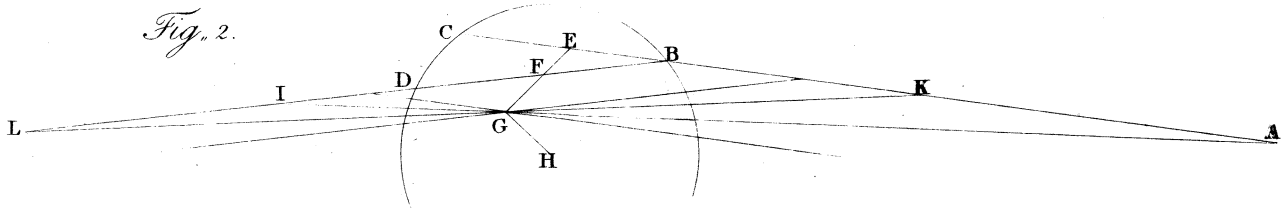

Fig. 3.

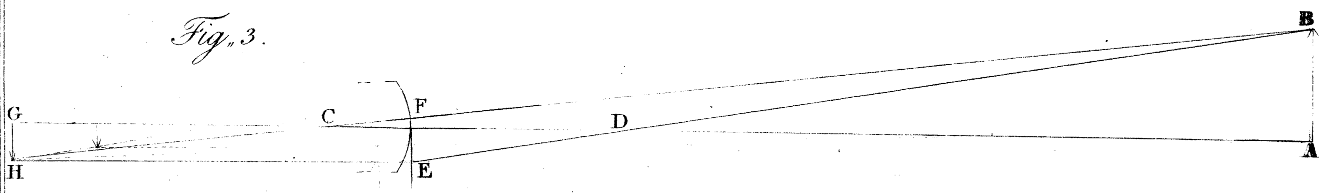

Fig. 4.

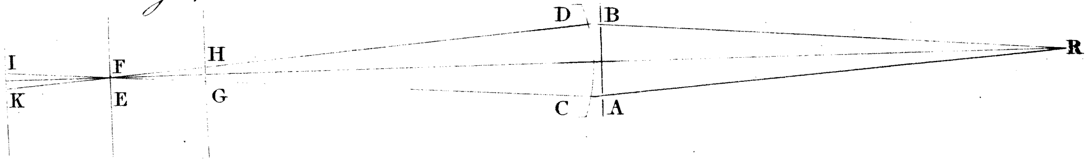

Fig. 5.

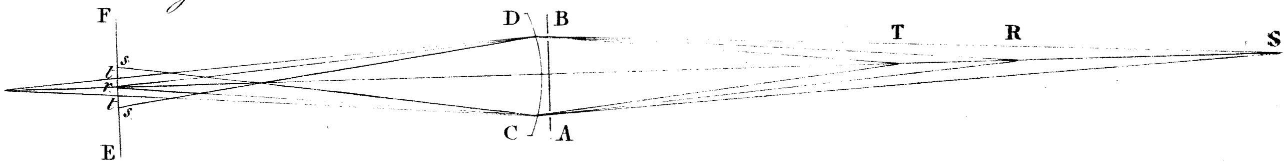

Fig. 6.

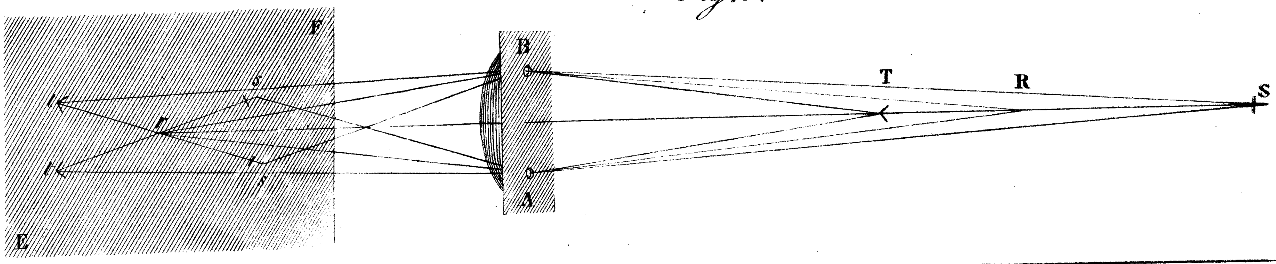

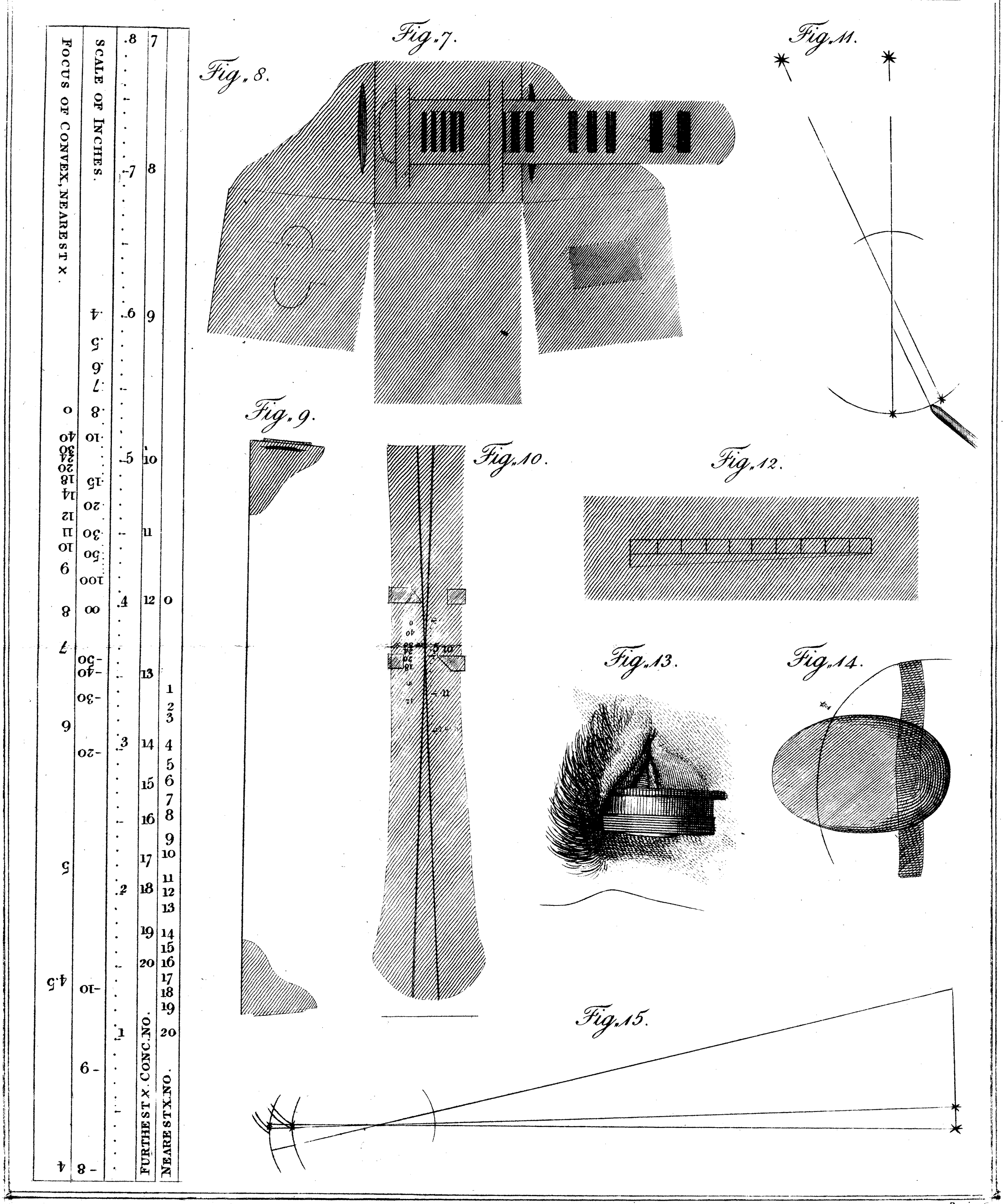
Fig. 7.
Fig. 8.
Fig. 9.
Fig. 10.
Fig. 11.
Fig. 12.
Fig. 13.
Fig. 14.
Fig. 15.
SCALE OF INCHES.
FOCUS OF CONVEX, NEAREST X.
FURTHEST X. CONC. NO.
NEAREST X. NO.

Basire sc.

Fig. 16.

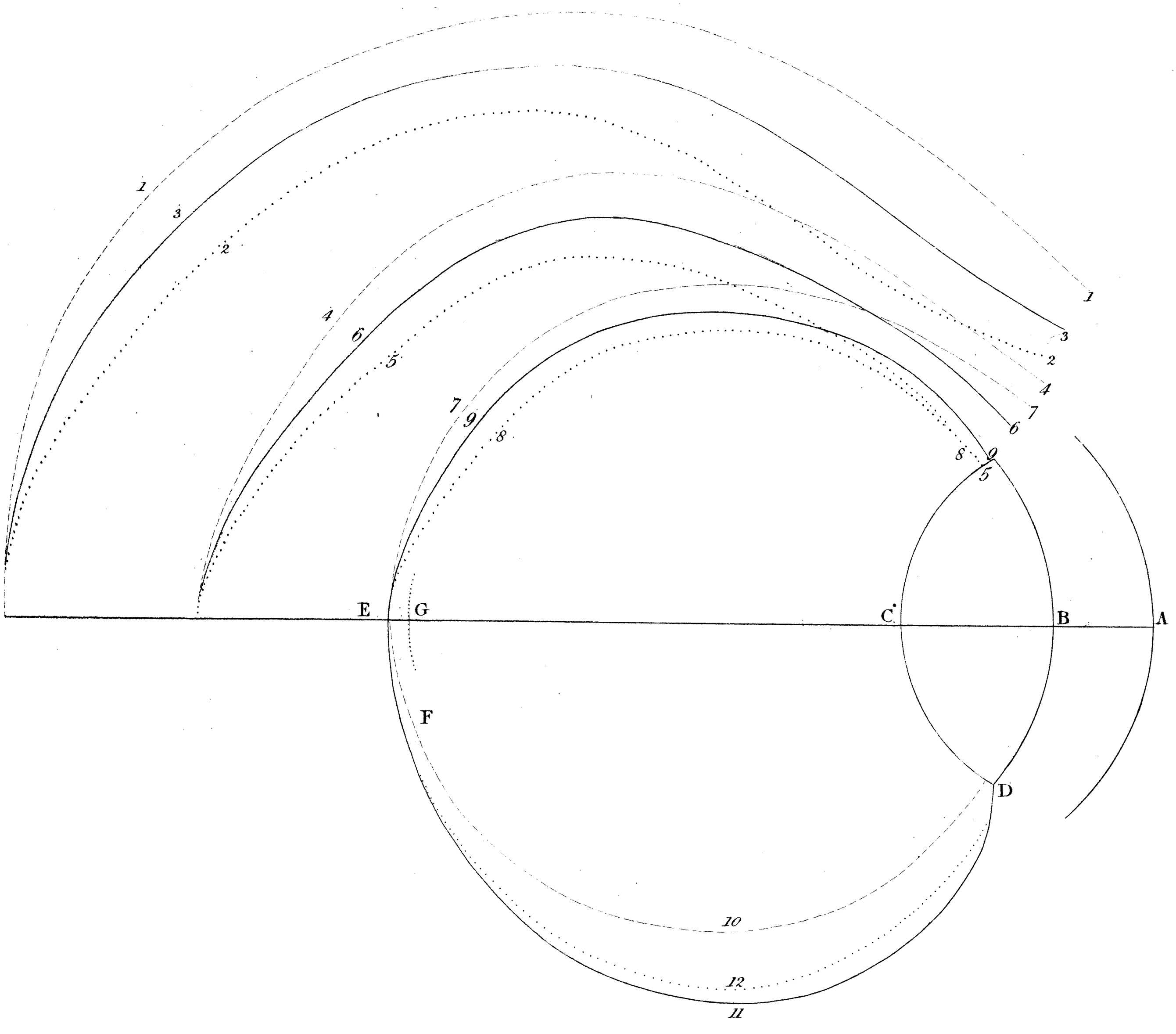

Basire sc.

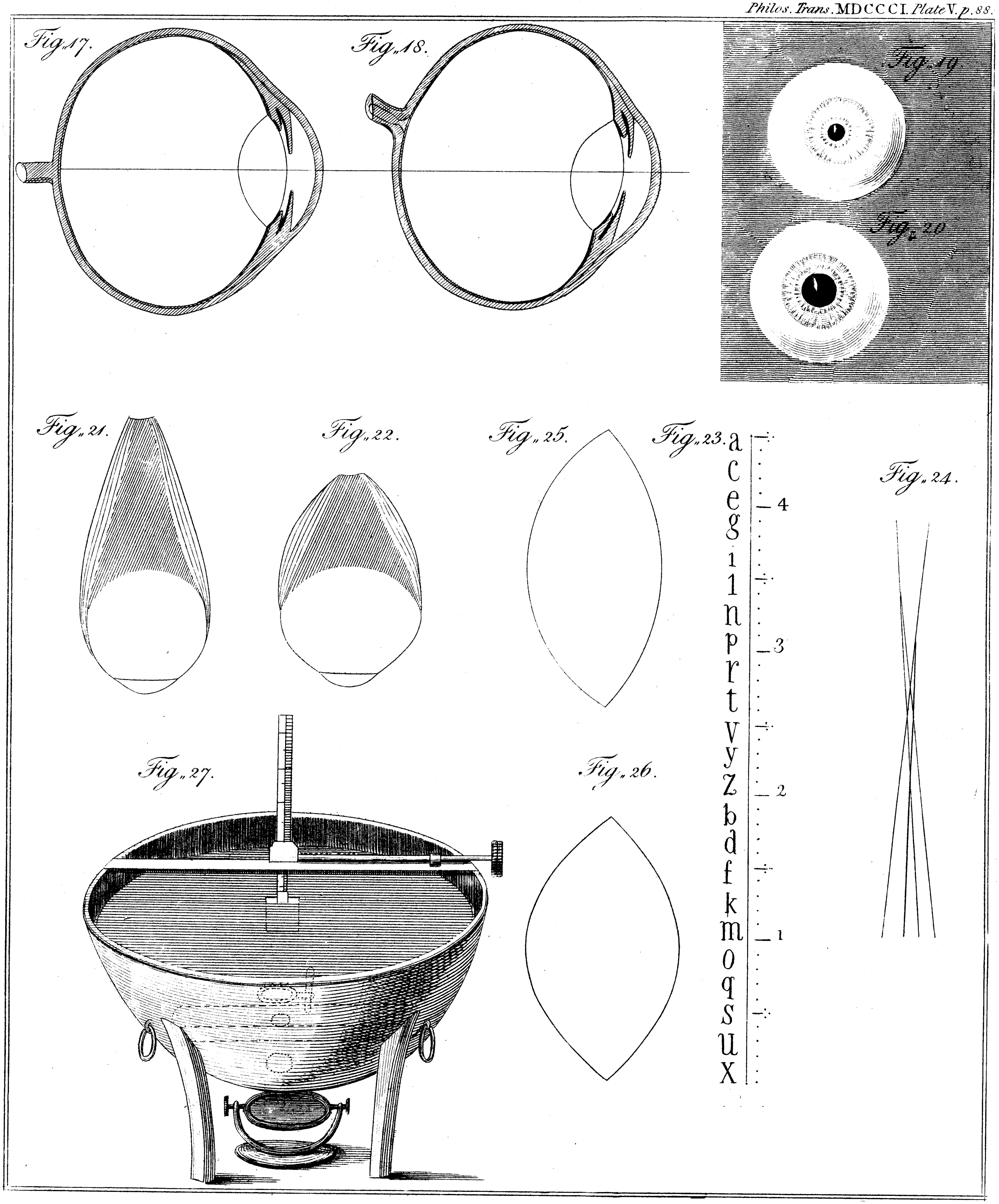
Fig. 17.
Fig. 18.
Fig. 19
Fig. 20
Fig. 21.
Fig. 22.
Fig. 25.
Fig. 23.
a c e g i l n p r t v y z b d f k m o q s u x
4
3
2
1
Fig. 24.
Fig. 27.
Fig. 26.

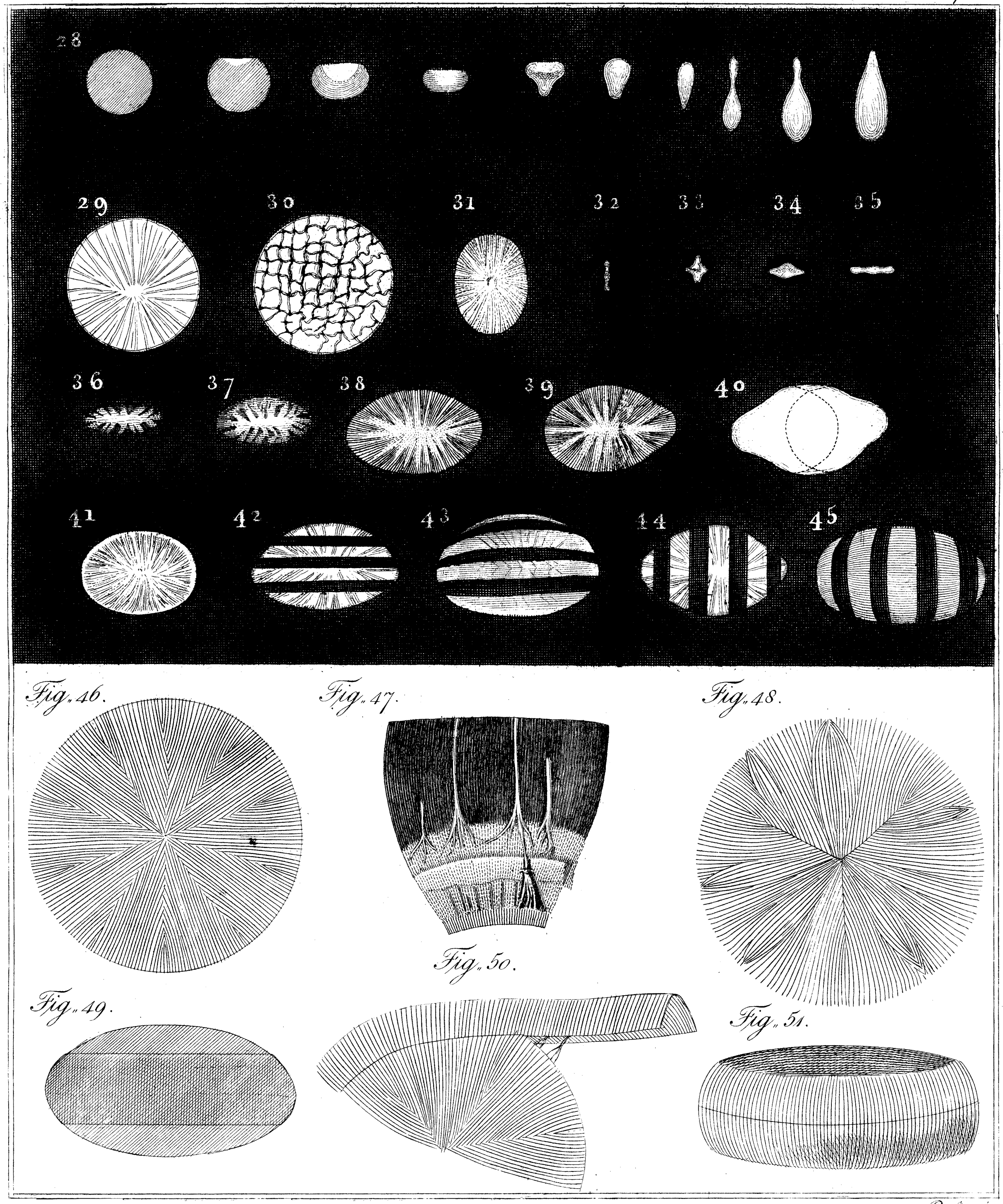
28
29
30
31
32
33
34
35
36
37
38
39
40
41
42
43
44
45
Fig. 46.
Fig. 47.
Fig. 48.
Fig. 50.
Fig. 49.
Fig. 51.

Basire sc.

Fig 52.

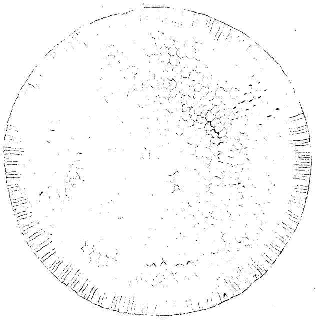

Fig 53.

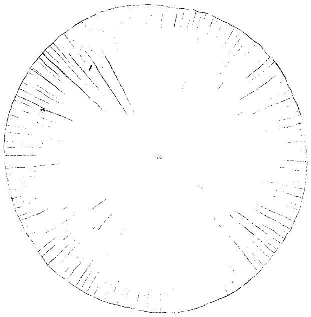

Fig 54.

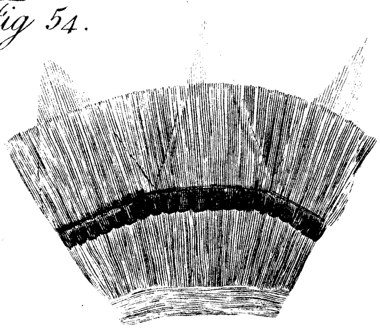

Fig 55.

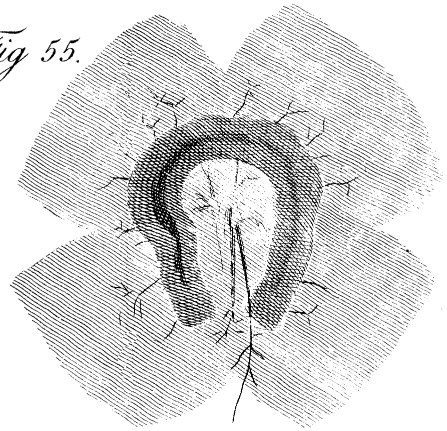

Fig 56.

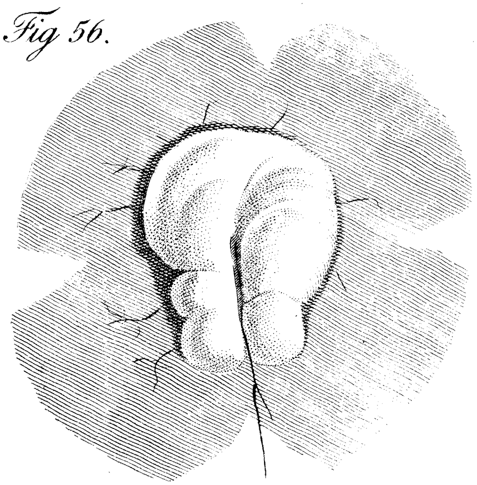

Fig 57.

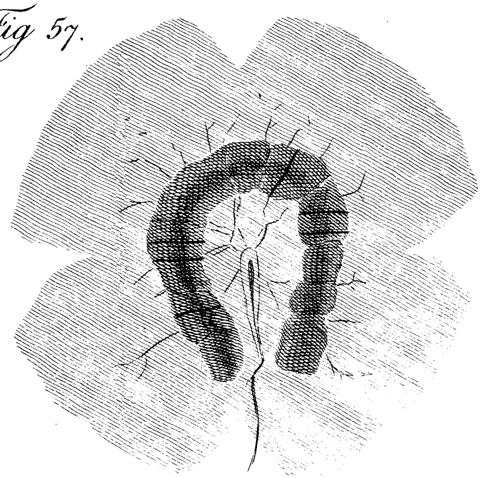

Basire sc.
